# Phenotypic variability and neuropsychological findings associated with *C9orf72* repeat expansions in a Bulgarian dementia cohort

**DOI:** 10.1371/journal.pone.0208383

**Published:** 2018-12-14

**Authors:** Shima Mehrabian, Håkan Thonberg, Margarita Raycheva, Lena Lilius, Katya Stoyanova, Charlotte Forsell, Lena Cavallin, Desislava Nesheva, Eric Westman, Draga Toncheva, Latchezar Traykov, Bengt Winblad, Caroline Graff

**Affiliations:** 1 Depatment of Neurology, UH “Alexandrovska”, Medical University-Sofia, Sofia, Bulgaria; 2 Karolinska Institutet, Dept NVS, Division for Neurogeriatrics, Bioclinicum, Akademiska stråket, Solna, Sweden; 3 Karolinska University Hospital, Theme Aging, Genetics Unit, Solna, Sweden; 4 Karolinska Institutet, Department of Clinical Neuroscience, Karolinska University Hospital, Department of Radiology, Stockholm, Sweden; 5 Department of Genetics, Medical University-Sofia, Sofia, Bulgaria; 6 Karolinska Institutet, Department of Neurobiology, Care Sciences and Society (NVS), Center for Alzheimer Research, Division of Clinical Geriatrics, Neo, Huddinge, Sweden; 7 Karolinska University Hospital, Theme Aging, Clinical Trial Unit, Stockholm, Sweden; 8 Karolinska Institutet, Department NVS, Center for Alzheimer Research, Division of Neurogeriatrics, Huddinge, Sweden; Oslo Universitetssykehus, NORWAY

## Abstract

**Background:**

The GGGGCC repeat expansion in the *C9orf72* gene was recently identified as a major cause of amyotrophic lateral sclerosis (ALS) and frontotemporal dementia (FTD) in several European populations. The objective of this study was to determine the frequency of *C9orf72* repeat expansions in a Bulgarian dementia cohort and to delineate the associated clinical features.

**Methods and findings:**

PCR-based assessments of the *C9orf72* hexanucleotide repeat expansion in all study samples (including 82 FTD, 37 Alzheimer’s disease (AD), and 16 other neurodegenerative/dementia disorder cases) were performed. We report the clinical, neuropsychological, and neuroimaging findings obtained for the *C9orf72* repeat expansion carriers. Of the 135 cases screened, 3/82 (3.7%) of all FTD cases and 1/37 (2.7%) of all clinical AD cases had a *C9orf72* repeat expansion. In this cohort, the *C9orf72* pathological expansion was found in clinical diagnoses bridging the FTD, parkinsonism, ALS and AD spectrum. Interestingly, we showed early writing errors without aphasia in two subjects with *C9orf72* expansions.

**Conclusions:**

This study represents the first genetic screening for *C9orf72* repeat expansions in a Bulgarian dementia cohort. The *C9orf72* repeat expansion does not appear to be a common cause of FTD and related disorders. This report confirms the notion that *C9orf72* repeat expansions underlie a broad spectrum of neurodegenerative phenotypes. Relatively isolated agraphia in two cases with *C9orf72* repeat expansions is a strong motivation to provide detailed and sophisticated oral and written language assessments that can be used to more precisely characterize early cognitive deficits in these heterogeneous conditions.

## Introduction

Recently, expansion of a GGGGCC hexanucleotide repeat in the gene *C9orf72* has been identified as the most common genetic cause of frontotemporal dementia (FTD) and amyotrophic lateral sclerosis (ALS), two diseases that belong to the general class of disorders referred to as c9FTD/ALS [1,2,3]. Several mechanisms, including RNA toxicity, repeat-associated non-AUG translation-mediated dipeptide protein aggregation, and haploinsufficiency of *C9orf72*, are suggested to be implicated in the molecular pathogenesis of these disorders [4].

Mutations in the *C9orf72* gene are associated with a spectrum or continuum of clinical manifestations with isolated FTD at one end, motor neuron disease (MND) at the other and a combination of behavioural/cognitive and MND symptomatology in between. However, expansions have also been reported in clinical and pathologically confirmed cases of Alzheimer’s disease (AD) and rarely in Parkinson’s disease, Creutzfeldt-Jakob disease, Huntington’s disease, slowly progressive behavioural variant FTD (bvFTD), pathologically confirmed dementia with Lewy bodies, corticobasal degeneration (CBD), ataxic syndromes and progressive supranuclear paralysis (PSP) [5,6,7,8,9,10,11,12]. The reported frequency of *C9orf72* repeat expansion in various populations is 23% to 50% in familial ALS cases, with a possible north-south descending gradient in Europe, and 4% to 8% in individuals with sporadic ALS [13,14,15,16]. Similar mutational frequencies have been described in patients with familial (15% to 55%) or sporadic (2%-6%) FTD [12,17,18]. A Slovenian study of patients in Eastern/Central European countries detected a rate of 5.9% pathogenic *C9orf72* expansions among ALS patients (5 of 85 cases) [19], and recently in a cohort of Serbian patients with early-onset dementia, the *C9orf72* hexanucleotide expansion was detected in 4 of 117 (3.4%) patients [20]. The study reported here represents the first genetic screening of *C9orf72* repeat expansions in a Bulgarian cohort of FTD/AD and related disorders. We report the detailed clinical, neuropsychological, and neuroimaging findings for four *C9orf72* repeat expansion carriers with a broad phenotypic spectrum of neurodegenerative disease.

## Materials and methods

### Clinical examinations

Given the growing evidence of genetic and clinicopathologic overlap in neurodegenerative diseases, we considered all subtypes of FTD, early-onset AD (EOAD) (age at onset before 65 years; familial and/or atypical cases), autosomal dominant late-onset AD (LOAD), other familial/early-onset neurodegenerative/dementia disorders, and consent to genetic analysis as inclusion criteria for this study. The study sample consists of consecutive cases who were seen from 2012–2014 at the Department of Neurology in Sofia, Bulgaria and who consented to participation in the study. The study cohort comprised 82 patients with different FTD subtypes, 32 EOAD (familial EOAD and/or EOAD with atypical clinical feature) patients, 5 autosomal dominant (LOAD) cases and 16 patients with other mixed early-onset/familial neurodegenerative/dementia disorders. The FTD spectrum disorders included 42 bvFTD cases, 12 non-fluent primary progressive aphasia (nf-PPA) cases, 5 patients with semantic variant of PPA and 7 FTD-ALS patients (including six cases of bvFTD-ALS and one nf-PPA-ALS patient). Thirteen patients received a clinical diagnosis of corticobasal syndrome (CBS), and 3 patients were diagnosed with PSP overlapping with FTD spectrum disorder. Other mixed early-onset/familial neurodegenerative disorders included early parkinsonian syndrome (six patients), spinocerebellar ataxia (SCA) (two patients), multiple system atrophy (MSA) (two patients), and autosomal dominant dementia with leukoencephalopathy and intracerebral haemorrhage (six patients).

The diagnosis of AD was made according to the NINCDS-ADRDA criteria [21]. A diagnosis of bvFTD, semantic-variant PPA, nf-PPA [22,23], FTD-ALS, PSP and CBS was considered based on current diagnostic guidelines [24,25,26]. Patients were evaluated using a standard protocol that included a detailed clinical and family history, neurological examination, comprehensive neuropsychological assessment, and neuroimaging. Family history was acquired by interviewing a knowledgeable informant. The disease was considered dominant if at least 3 individuals in two or more generations suffered from early-onset dementia and two of the individuals who suffered from early-onset dementia are first-degree relatives of the third. When the criteria for dominant inheritance were not fulfilled, the disease was considered familial if at least two individuals (third-degree relatives or closer) suffered from the disease. Sporadic cases were determined as the presence of one sporadic case or when the relatives with dementia were third-degree or more distant relatives. Unknown heredity was indicated when insufficient information about the family was available, e.g., due to adoption, deaths of family members at an early age or no known family history.

All participants or their caregivers gave written informed consent to participation in the clinical and genetic studies and to brain biopsy where appropriate. The individuals in this manuscript have given written informed consent (as outlined in PLOS consent form) to publish these case details. The informed consent forms and study protocols were approved by the local ethics committee, Stockholm, and the local ethics committee, Medical University, Sofia, and performed in harmony with the Helsinki Declaration.

### Neuropsychological assessments

The patients underwent comprehensive neuropsychological assessment using a test battery designed to evaluate several cognitive domains (memory, gnosis, praxis, language, and attention/executive functions). We assessed global cognitive functioning using the standard Mini-Mental State Examination (MMSE; maximal score 30 points) [27]. Learning and episodic verbal memory were assessed using the Buschke Free and Cued Selective Reminding Test (FCSRT) [28]. Attention was tested using the Trail-Making Test part A (TMT-A), which provides a timed measure of selective attention to visually presented information. Cognitive flexibility as part of executive function was tested using the Trail-Making Test part B (TMT-B), which assesses the time taken by the patient to correctly relay all items in each of the trials [29]. Further tests of executive function included the Stroop Color Word Test (SCWT) [30], verbal fluency (Isaac’s Set Test, IST) [31], and phonemic verbal fluency (number of words beginning with the letter M produced in 60 seconds). Language abilities were assessed based on the 15-item subset of the Boston Naming Test (BNT) [32], semantic verbal fluency (names of animals produced in 60 seconds) [29], the Boston Diagnostic Aphasia Examination (BDAE) subtests [33], semantic tasks (word-to-picture discrimination, semantic association), and the Picture Naming Object Test (60 items) [34]. Written language abilities were assessed using spontaneous writing tasks, a picture description task (cookie theft task, BDAE), written naming (15 items, BNT), writing from dictation, and writing from copy. The Rey-Osterrieth Complex Figure Test (ROCFT) [35] and the figure copy sub-test (CERAD Neuropsychological Battery) were used to assess visuospatial abilities and constructional praxis. Non-verbal memory was examined using delayed recall of the ROCFT and by the figure copy test (CERAD Neuropsychological Battery) [36]. The digit span test (forward and backward) was performed to assess short-term and working memory [35].

Core behavioural symptoms of FTD were systematically explored during an interview with each patient’s caregiver. The interview was conducted by a neurologist or a neuropsychologist and recorded on a standardized inventory based on the Frontal Behaviour Inventory (FBI). FBI is a quantitative caregiver-based scale that consists of 24 behavioural and personality items designed to probe the core behavioural features of FTD [37]. The conservative cut-off FBI score is above 27 for FTD, with a maximum possible FBI score of 72. The geriatric depression scale (GDS) [38] was also assessed.

### Neuroimaging

Patients underwent T1- and T2-weighted magnetic resonance imaging (MRI, 1.5 T and 3 T). The following previously validated MRI visual rating scales were used and described for the patients with *C9orf72* repeat expansions: the five-step Kipps/Davies scale for frontotemporal atrophy [39]; the four-step visual rating scale for posterior brain regions [40]; the four-step (generalized) Pasquier scale for global cortical atrophy [41]; the five-step Scheltens scale for medial temporal atrophy [42]; and the Fazekas scale for white matter hyperintensities (WMH) with four severity grades [43]. Areas of atrophy were rated by a neuroradiologist who was blinded to the diagnosis.

### Molecular analysis

Genomic DNA was isolated from peripheral blood according to standard protocols. The quality and quantity of DNA were assessed by fluorometry. All DNA samples were diluted to a final concentration of 200 ng/mL.

### *C9orf72* G_4_C_2_ Genotyping assays

Repeat-primed PCR (RP-PCR) and a short tandem repeat (STR) fragment length assay were used to analyse the *C9orf72* G_**4**_C_**2**_ expansion according to a previous report but with a minor modification in which 20 μM 7-deaza-dGTP was added to the RP-PCR reaction [44,45]. The presence of more than 40 G_**4**_C_**2**_ repeats was considered pathogenic. All sequences, repeat-primed PCR and STR products were run on an ABI 3100 Genetic Analyzer. The primer sequences and PCR conditions are available upon request. The *C9orf72* G_**4**_C_**2**_ genotyping assays were performed at the Karolinska Institutet, Huddinge, Sweden.

## Results

This cohort (135 dementia patients) included 29 cases with family histories that were compatible with autosomal dominant inheritance, 48 familial cases, 42 sporadic cases and 16 individuals with unknown inheritance. There was a significantly higher proportion of both dominant and familial cases in EOAD than in FTD (the majority of them were early-onset FTD). The mean age at onset of the cohort was 55.9±9.0 years, ranging from 23 to 77 years.

We found that 27 individuals of this cohort (20%) had age at onset ≤50. The majority of individuals had a diagnosis of FTD, predominantly bvFTD. Other diagnoses included EOAD, parkinsonian syndrome and autosomal dominant dementia with leukoencephalopathy and intracerebral haemorrhage.

The clinical characteristics of the patients are presented in Table 1.

**Table 1:** Clinical characteristics of the study population.

Study samples Patients (N = 135)	FTD (N = 82)	EOAD (N = 32)	Other diagnosis* (N = 21)
Gender (female/male)	48/34	18/14	9/12
Age at onset	56.7±7.3	54 ±11.1	55.8±17.8
Dominant cases (%)	10 (12.2%)	12 (37.5%)	7 (33.3%)
Familial cases (%)	33 (40.2%)	9 (28.1%)	6 (28.6%)
Sporadic cases (%)	30 (36.6%)	7 (21.9%)	5 (23.8%)
Unknown cases (%)	9 (11.1%)	4 (12.5%)	3 (14.3%)

FTD–frontotemporal dementia (FTD group includes all subtypes of FTD); EOAD–early-onset Alzheimer’s disease.

^*^the ‘other diagnosis’ group includes 16 other familial/early-onset neurodegenerative/dementia disorders and 5 autosomal dominant late onset-Alzheimer’s disease (LOAD) cases.

### Frequency of *C9orf72* repeat expansions

Of the 135 cases screened, four were found to have the *C9orf72* repeat expansion. All four subjects were heterozygous for the expansion mutation. No mutations were identified in the 16 screened cases of neurodegenerative disorders other than AD and FTD spectrum. In summary, 3/82 (3.7%) of all FTD cases and 1/37 (2.7%) of all clinical AD cases had a *C9orf72* repeat expansion.

The observed range of sizes of the second, wild-type, allele was 2–19 units; the most frequent repeat size was 2 units, followed by 5, 8, and 6 in that order. In expansion carriers, we did not observe an intermediate or pathological number of repeats for the second wild-type allele (2–6 repeats).

**Fig 1:**
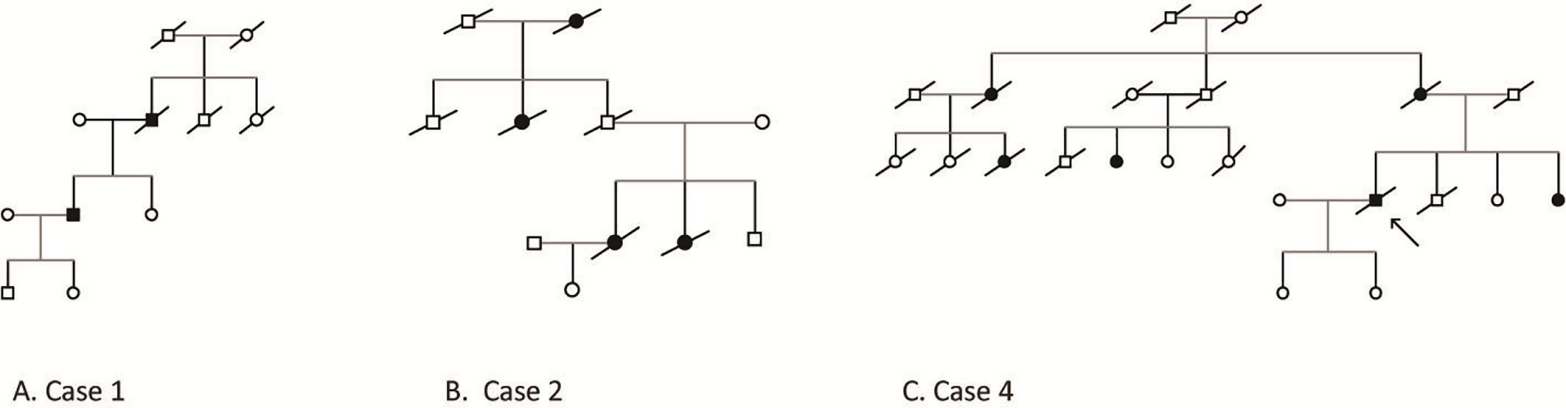
Family trees of *C9orf72* expansions in the Bulgarian cohort (A. Case 1, B. Case 2, C. Case 4).

Two index patients with *C9orf72* expansion mutations had family histories that were compatible with autosomal dominant inheritance; one patient had at least one affected relative, but the criteria used for autosomal dominant inheritance were not met. One expansion carrier had no family history of dementia. The family trees of probands 1–4 are shown in Fig 1.

### Clinical, behavioural and neuroimaging features

The demographic data for the four *C9orf72* expansion carriers are summarized in Table 2. The average age at onset for the *C9orf72* expansion carriers was 52.5±5.6 (±SD, range 49–61) years. Mean disease duration from symptom onset until death (n = 3) or time of last review (n = 1) was 7.2±3.6 (±SD, range 4–12) years.

**Table 2:** Clinical characteristics of patients with *C9orf72* expansions.

Characteristics	Case 1	Case 2	Case 3	Case 4
Clinical diagnosis	bvFTD	FTD/ALS	FTD-PSP	Probable AD
Disease duration (years)	12	4	5	8
Hereditary	Familial	Dominant	Sporadic	Dominant (mix onset)
Debut symptom	Behavior changes, anxiety	Apathy, bradykinesia	Disorganization falls, behavior changes	Memory impairment
**Neurological exam** • Brisk reflexes	+	+	+	+
• Hoffman	-	+	-	+
• Babinski	-	+	-	-
• Frontal release signs	+	+	+	+
• Pseudobulbar signs	+	+	+	-
• Bulbar signs	-	+	-	-
• Rigidity	+	+	+	+
• Bradykinesia	+	+	+	+
• Postural instability	-	+	+	-
• Tremor	-	-	-	-
**UPDRS–III**	18	27	44	14
**Dopa-responsive**	+	-	-	-
**GDS**	4	5	4	4

bBvFTD–behavioural variant of frontotemporal dementia; PSP–progressive supranuclear paralysis; ALS–amyotrophic lateral sclerosis; AD–Alzheimer’s disease; UPDRS–unified Parkinson's disease rating scale; GDS–Geriatric Depression Scale.

Clinical phenotypes varied across the four patients with *C9orf72* repeat expansions. One patient was classified clinically as having bvFTD, one patient as having FTD-ALS, one as FTD-PSP overlap and one as having clinically probable AD. All four patients were at a relatively early stage in the course of cognitive decline at the time neuropsychological testing was performed; thus, the early neuropsychological profile of impairment was observed. The data on the individual patients are described in detail below and are presented in Tables 2–5. Motor symptoms were examined using Part III of the Unified Parkinson`s Disease Rating Scale (UPDRS). The neurological signs and behavioural symptoms of the four patients are described in more detail in Tables 2 and 3.

**Table 3:** Core behavioural features of patients with *C9orf72* expansions.

Frontal behavior Inventory (FBI) (Kertesz, et al, 2000)	Case 1 (bvFTD) After 1 y		Case 2 (FTD/ALS) After 1 y		Case 3 (FTD-PSP)	Case 4 (AD) After 2 y	
**MMSE**	20	15	26	24	25	23	14
**FBI-A**	18	25	15	21	21	5	18
• Apathy	3	3	3	3	2	1	2
• Aspontaneity	3	3	3	3	2	1	2
• Indifference	1	2	1	2	2	0	2
• Inflexibility	2	3	2	3	3	1	2
• Personal neglect	2	3	1	2	3	0	2
• Disorganization	3	3	2	3	3	1	3
• Inattention	1	2	2	2	3	1	2
• Loss of insight	2	3	1	2	2	0	2
• Logopenia	1	3	0	1	1	0	1
• Semantic dementia	0	0	0	0	0	0	0
• Aphasia	0	0	0	0	0	0	0
• Apraxia	0	0	0	0	0	0	0
FBI-B	14	25	5	8	17	2	10
• Perseveration	2	2	3	3	3	1	1
• Irritability	1	3	0	1	0	1	2
• Excessive jocularity	0	0	0	0	0	0	0
• Impulsivity	3	3	1	2	2	0	0
• Hoarding	1	3	0	0	0	0	0
• Inappropriateness	2	3	1	1	3	0	1
• Roaming	1	3	0	0	0	0	2
• Aggression	1	2	0	0	0	0	1
• Hyperorality	1	2	0	0	2	0	2
• Hypersexuality	0	0	0	0	2	0	0
• Utilization behavior	1	1	0	0	2	0	0
• Incontinence	1	3	0	1	3	0	1
**FBI–total**	**32**	**50**	**20**	**29**	**38**	**7**	**28**

bBvFTD–behavioural variant of frontotemporal dementia; PSP–progressive supranuclear paralysis; ALS–amyotrophic lateral sclerosis; AD–Alzheimer’s disease; MMSE–Mini Mental State Examination.

**Table 4:** Visual rating scales (MRI) of patients with *C9orf72* expansions.

Visual rating scales (MRI) (right/left)	Case 1 (bvFTD)	Case 2 (FTD/ALS)	Case 3 (FTD-PSP)	Case 4 (AD)
**MMSE (at the time of neuroimaging)**	20	26	25	23
**MTA atrophy (0–4)** **(**Scheltens P, 1992)	2/2	1/1	2/2	2/2
**Fazekas scale (0–3)** **(**Fazekas F, 1987)	2	0	1	1
**Posterior Atrophy (0–3) (Koedam, 2011)**	0/0	0/0	0/0	2/2
**Lobar atrophy (Kipps, 2007**				
• **Anterior temporal lobe (0–4)**	2/2	1/0	0/0	2/2
• **Posterior temporal lobe (0–4)**	2/2	0/0	0/0	2/2
**Global Cortical Atrophy (0–3)** (Pasquier F, 1996)				
• **Frontal** **Ventricular dilatation**	2/2	1/1	0/0	1/1
• **Frontal**	1/1	1/1	1/1	1/1
• **Parieto-occipital**	1/1	1/1	1/1	1/1
• **3-rd ventricle**	2	0	1	1

MRI–magnetic resonance imaging; bvFTD–behavioural variant of frontotemporal dementia; PSP–progressive supranuclear paralysis; ALS–amyotrophic lateral sclerosis; AD–Alzheimer’s disease. Mild frontal atrophy asymmetry that could not be considered in the visual scale scores was noted in Cases 1 and 2.

**Table 5:** Neuropsychological assessment of patients with *C9orf72* expansions.

Neuropsychological features	Case 1	Case 2	Case 3	Case 4
Clinical diagnosis	bv-FTLD	FTD/ALS	FTD-PSP	Probable AD
MMSE	20/30	26/30	25/30	23/30
Verbal Episodic memory Buschke (FCSRT)				
• Free recall	20/48	19/48	31/48	14/48
• Total recall (free+cued)	30/48	43/48	45/48	26/48
• Delayed free recall	2/16	6/16	10/16	4/16
• Total delayed recall (free+cued)	4/16	10/16	16/16	10/16
• Recognition	40/48	45/48	46/48	36/48
Non-verbal memory				
• Rey–Osterrieth complex figure test (ROCF)–delayed recall	-	-	21/36	-
• Copy figure test (CERAD)delayed recall	5/11	7/11	-	4/11
Short-term memory				
• Digit span forward	5	7	7	6
Working memory				
• Digit span backward	3	4	4	4
Attention/Executive functions				
• TMT-A	215 (2 errors)	136 (1 error)	NA	127
• TMT-B	NA	360 (5 errors)	NA	245 (3 errors)
• Stroop test				
I	51	40	NA	64
II	31 (1 error)	29 (2 errors)	NA	55
III (incongruent)	15 (5 errors)	17 (4 errors)	NA	31 (3 errors)
Verbal fluency				
• Phonetic (M)	1	2	3	7
• IST	12	21	22	25
Vasoconstrictive abilities • Rey–Osterrieth complex figure test (ROCF) Copy figure test (CERAD)	- 9/11	- 10/11	31/36 -	- 7/11
Language				
• BNT (oral)	13/15	13/15	13/15	14/15
• BNT (written)	3/15	7/15	15/15	15/15
• BDAE–comprehension/commands	13/15	14/15	-	11/15
• BDAE—Paragraph comprehension	-	-	9/12	-
• BDAE–reading comprehension (words)	8/8	8/8	8/8	8/8
• Verbal Fluency Semantic	6	11	9	15
Apraxia	mild (ideomotor, symbolic)	-	-	-
Agraphia	+ (agraphia)	+ (agraphia)	Micrographia	-

NA–not applicable; bvFTD–behavioural variant of frontotemporal dementia; PSP–progressive supranuclear paralysis; ALS–amyotrophic lateral sclerosis; AD–Alzheimer’s disease; FCSRT–Free and Cued Selective Reminding Test; CERAD–Consortium to Establish a Registry for Alzheimer's Disease; TMT–Trail-Making Test; BDAE–Boston Diagnostic Aphasia Examination; IST–Isaacs Set Test.

The MRI visual rating scores revealed variability across the cases. The group data obtained using the MRI visual rating scales for the four C9 cases are summarized in Table 4.

### CASE 1 (bvFTD)

**Fig 2:**
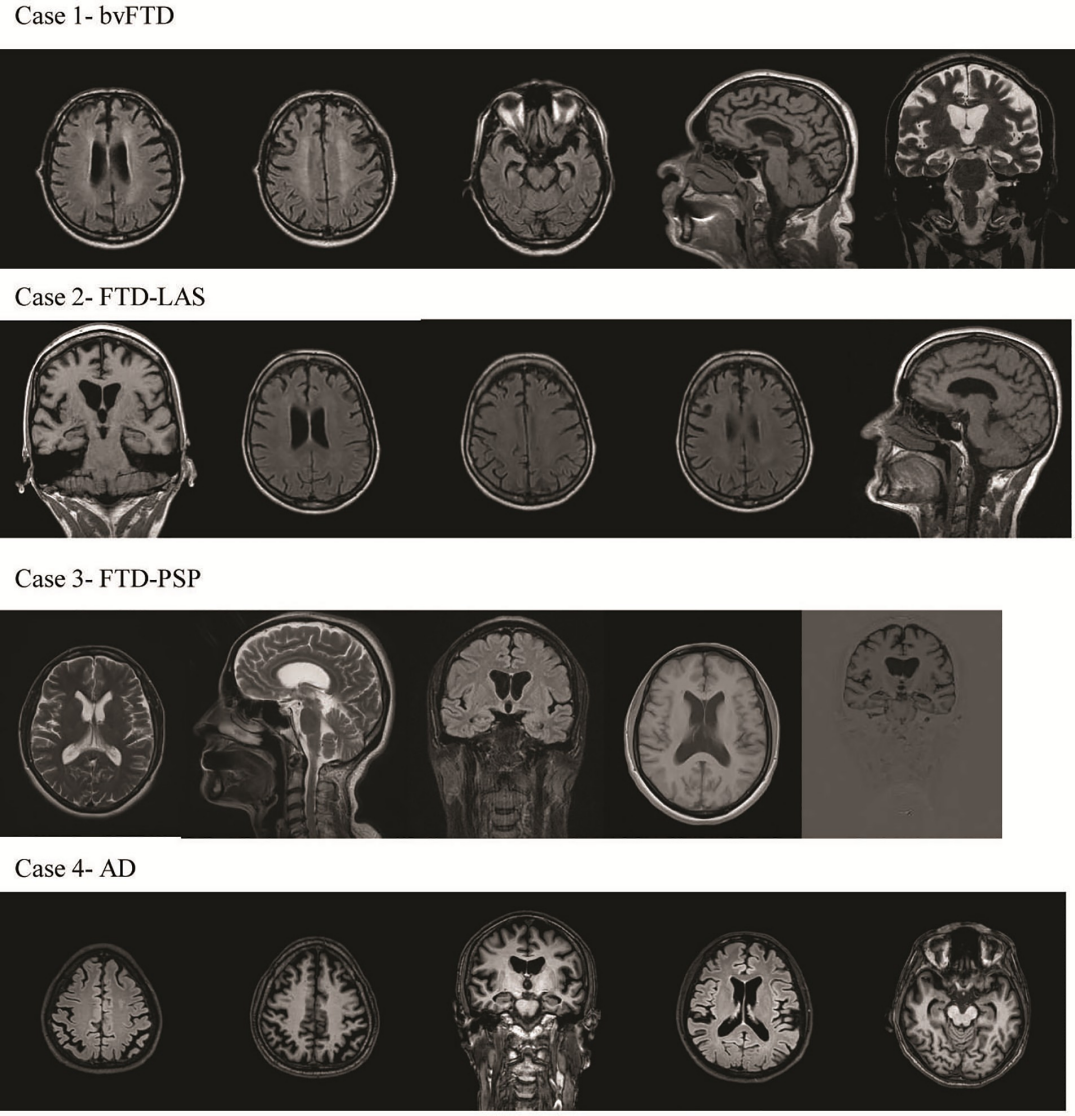
Brain MRI (T1, T2 and Flair) of *C9orf72* expansions in the Bulgarian cohort (Case 1, 2, 3, 4). Case 1—Bilateral frontal and temporal atrophy with very mild asymmetry that was more pronounced in the left hemisphere and hippocampal atrophy; Case 2—Mild frontal atrophy with very mild asymmetry that was more pronounced in the left frontal lobe and very mild hippocampal atrophy; Case 3—Mild hippocampal atrophy; Case 4—Generalized cortical atrophy, including posterior atrophy and bilateral hippocampal atrophy.

At the age of 50, the patient, a male with secondary education (11 years of total education), displayed subtle behavioural changes, including anxiety and sleep disturbances, at least seven years before bvFTD was diagnosed. Six years after his initial symptoms, he was hospitalized at the psychiatry clinic due to the presence of neuropsychiatric symptoms (delusions and auditory and visual hallucinations). He became irritable and often came into conflict with his neighbours. Neuroleptic therapy was started. Apathy, aspontaneity, social isolation and loss of insight were also noticed by the family. The patient had relatively preserved general cognition (MMSE = 27). At that time, he was laid off from work owing to conflict, aggressive behaviour and delusions. One year later, on neurological examination, he had brisk reflexes, frontal release signs, mild dysarthria and dopa-responsive parkinsonism with rigidity and bradykinesia. Brain MRI showed bilateral frontal and temporal atrophy with very mild asymmetry that was more pronounced in the left hemisphere and hippocampal atrophy (Fig 2).

Neuropsychological examination revealed mild to moderate dementia (MMSE = 20). The patient showed verbal episodic memory impairment of predominantly hippocampal type. He exhibited pronounced dysexecutive symptoms and mild limb apraxia (ideomotor, symbolic, and constructive) (Table 5).

**Fig 3:**
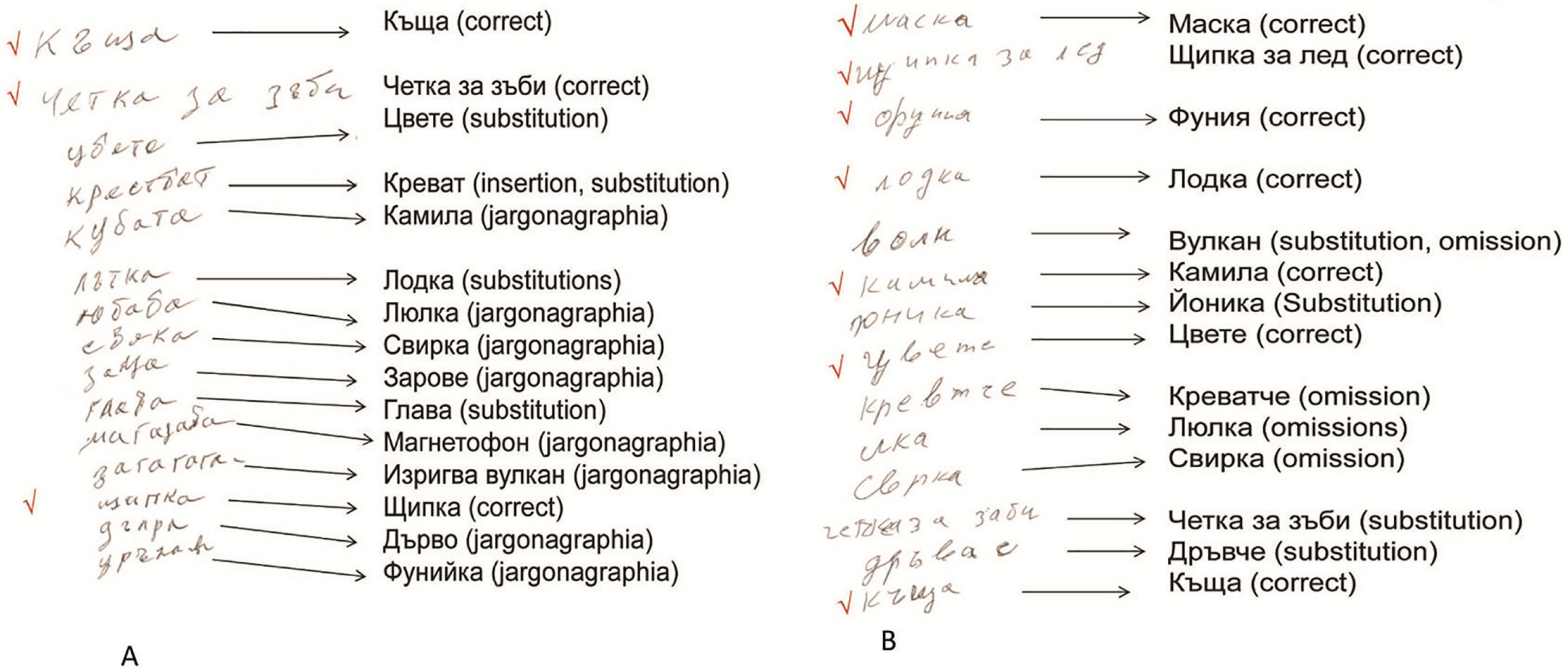
Writing errors in two patients with *C9orf72* expansions. A. Case 1 (bvFTD diagnosis). B. Case 2 (FTD-ALS diagnosis).

On language examination, there were no signs of aphasia either in oral language expression or in auditory comprehension. The patient’s confrontation naming was relatively preserved (BNT = 13/15). In written naming of the same stimuli, he showed pronounced agraphia (BNT = 3/15), as well as in writing on dictation. Substitution of letters was the most frequent error. Transpositions, omissions, and insertion/repetition of letters, together with jargonographia (incomprehensible words), were observed. Nonverbal visuospatial disposition was preserved. We conclude that the patient had agraphia without aphasia (Table 5 and Fig 3).

The patient displayed disinhibition, perseveration, impulsivity, a tendency to overeat, and lack of empathy and insight. The FBI score was 32. Based on these clinical, behavioural, neuropsychological and neuroimaging findings, we arrived at a diagnosis of bvFTD according to current consensus criteria (Rascovsky et al., 2011).

One year later, we found progressive functional dependence, cognitive decline and worsened behaviour. The MMSE score decreased to 15. Spontaneous speech was reduced, with echolalia and more severe behavioural changes (FBI = 50). The patient’s overall behavioural symptoms improved after therapy with quetiapine. During the following 2 years, the disease progressed, resulting in mutism and severe dementia (MMSE = 0); extrapyramidal symptoms worsened, and the patient became bedridden.

The patient’s mother and sister have normal cognitive functioning at 85 and 57 years of age, respectively. The patient’s father experienced 5 years of progressive cognitive, behavioural and language disorder that progressed to death at age 70. The proband’s paternal grandfather and grandmother died early (in their 40s and 60s, respectively) (Fig 1).

### CASE 2 (FTD-ALS)

The patient, a female with secondary education (11 years of total education), initially presented at age 50 years with behavioural changes and slowness in movement. Apathy, loss of empathy, indifference, social isolation, perseveration and mild impulsivity and delusions were followed by the insidious development of ALS approximately one to two years after the onset of behavioural changes and movement slowness. The patient developed progressive dysarthria, dysphagia, and dysphonia. At the age of 52, neurological examination revealed mild bulbar and pseudobulbar signs, pyramidal signs with hyperreflexia and the presence of Hoffmann’s and Babinski signs bilaterally, spasticity in the extremities, distal amyotrophy of the upper extremities and extrapyramidal signs with non-dopa-responsive rigidity and bradykinesia. At this time, electromyographic findings in all four limbs and the tongue were normal, but intermittent tongue fasciculations were noted. Brain MRI revealed mild frontal atrophy with very mild asymmetry that was more pronounced in the left frontal lobe and very mild hippocampal atrophy (Fig 2). Neuropsychological examination demonstrated mild cognitive impairment (MMSE = 26). Moderate impairment of verbal episodic memory of predominantly hippocampal type (mildly impaired recognition and not completely normal recall with cueing) was observed. The patient exhibited pronounced executive impairment and relatively preserved visual constructive abilities without apraxia or aphasia (Table 5).

Neuropsychological examination of the patient’s written language revealed mild agraphia. Her confrontation naming was relatively preserved (BNT = 13/15). In written naming of the same stimuli, she showed agraphia (BNT = 7/15). Omission and substitution of letters were the most frequent errors (Fig 3). We conclude that the patient had mild agraphia without aphasia.

One year later, the presence of EMG in the tongue confirmed the diagnosis of bulbar ALS. The patient’s swallowing difficulties worsened, as did her articulation and parkinsonism. Neuropsychological examination revealed mild dementia (MMSE = 24) with mild difficulties in daily living activities. Mild progression in general cognition was detected. We observed worsening of dysarthria, agraphia, and acalculia as well as behavioural changes. The patient’s clinical status declined rapidly and she died at the age of 54, four years after symptom onset, of aspiration pneumonia.

The patient’s sister was affected by a clinically and electrophysiologically confirmed ALS phenotype with bulbar onset at the age of 41 years and without any apparent cognitive or behavioural impairment. Parkinsonism with rigidity and bradykinesia developed. She died two years after disease onset. The proband’s father died early at the age of 50 of lower extremity gangrene. In addition, a paternal aunt died of bulbar onset ALS in her 60s. A parental grandmother had behavioural impairment in her 70s with rapid progression of the disease to severe dementia (Fig 1).

### CASE 3 (FTD-PSP)

The patient is a female with higher education (16 years). Her problems were noticed at work at the age of 49. She began to make mistakes; became disorganized, perseverative, and apathetic; and was subsequently dismissed. She had frequent falls. Her house was chaotic and disorganized. Personal neglect, early urinary and foecal incontinence, tactlessness in social situations with little insight, and disinhibition were noticed. She had increased tobacco and food consumption and demonstrated sexual disinhibition. At the age of 52 years, hydrocephalus was diagnosed, and she underwent a ventriculoperitoneal shunting. After one year, she was referred to the memory clinic and recruited to this study. The neurological examination showed supranuclear vertical gaze palsy, limited saccadic eye movements, hypomimia, hypophonia, dysarthria, frontal release signs, micrographia, bradykinesia, axial rigidity, neck extension and postural instability with frequent falls. The patient was diagnosed with FTD-PSP overlap. Her extrapyramidal symptoms were non-dopa-responsive. The behavioural profile was consistent with bvFTD. Brain MRI showed only mild hippocampal atrophy (Fig 2).

Neuropsychological examination was performed under the condition of very limited eye movement due to the patient’s ophthalmoplegia. Mild dementia was recorded with MMSE = 25/30. Verbal and non-verbal learning was relatively preserved. Pronounced deficits in attention and executive functions were revealed. Reading, comprehension, naming and visual constructive abilities were relatively spared. In more sophisticated language assessments, very mild naming difficulties in the picture-naming object test (56/60) with four verbal paraphasias and mild difficulties in auditory comprehension of complex material were revealed. The patient had obvious micrographia without agraphic-type errors. The FBI score was 38. Neuropsychological profile revealed mild dementia dominated by behavioural and attentional/executive impairment. We conclude that the patient had FTD-PSP overlap.

During the following year, the patient became unable to move her eyes in a purposeful way and was bedridden because of very frequent falls. She died at the age of 54 years with very advanced parkinsonism and difficulty in eating but with relatively preserved memory and orientation. There were no known affected relatives. Both parents of the proband died at 58 years of age.

### CASE 4 (probable AD)

This male patient, with 11 years of education, presented initially at the age of 61 with a classical amnestic syndrome and subtle changes in executive function without any language, personality, or behavioural disturbances. Three years after the onset of disease, neurological, neuropsychological and neuroimaging examinations were performed. Neurological examination revealed brisk reflexes and mild extrapyramidal signs. Comprehensive neuropsychological examination showed mild dementia (MMSE = 23) with impairment in episodic memory of the hippocampal type and mild to moderate executive dysfunction, mild visuospatial deficit, and mild auditory comprehension deficit. Praxis, reading, naming and writing were relatively preserved. There was no sign of agraphia (Table 5). Mild apathy, aspontaneity and irritability were noticed. The Frontal Behaviour Inventory (FBI) score was 7. Brain MRI showed generalized cortical atrophy, including posterior atrophy and bilateral hippocampal atrophy (Fig 2). We conclude that this patient had probable EOAD.

During the following 2 years, a rapid decline was noted. Follow-up neurological examination demonstrated brisk reflexes with Hoffman sign, non-dopa-responsive extrapyramidal signs with rigidity and bradykinesia, and pseudobulbar signs. The patient had moderate dementia (MMSE = 14), reduced and stereotypic speech and decline in all cognitive domains. Behavioural changes such as hyperorality, roaming and aggression appeared, and the FBI was 28. Approximately 8 years after the initial presentation, the patient was bedridden and almost mute. He died at 69 years of age.

His sister and mother developed memory impairment and subsequent LOAD and died at age 71 and age 74, respectively. The proband’s maternal aunt and her daughter had progressive memory impairment and were diagnosed with EOAD. Behavioural impairment was noticed later in the disease course. A daughter of the proband’s uncle developed LOAD, and her brother, interestingly, died at age 50 with multiple sclerosis. The diagnosis was made by a neurologist on the basis of available brain MRI. The diagnosis of ALS is unlikely due to the disease course of more than 10–12 years. The proband’s maternal grandmother had cancer and died early in her 30s (Fig 1).

## Discussion

In this specialized dementia clinic cohort, we found that 3.7% of all FTD cases (including the FTD-ALS patients) and 2.7% of all clinical AD cases had a *C9orf72* repeat expansion, revealing a relatively low frequency of expanded repeats in the Bulgarian dementia cohort. The phenotypic spectrum of patients carrying *C9orf72* hexanucleotide repeat expansions was broad, even within the same family pedigree (in Case 2). We described four clinical histories associated with *C9orf72* expansions: bvFTD, FTD-ALS, FTD-PSP, and AD, thereby confirming the clinical heterogeneity of this syndrome also in the Bulgarian cohort. The clinical, behavioural, and neuropsychological (particularly memory and language domains) symptoms and the results of neuroimaging varied considerably in the mutation carriers.

In a pan-European study of FTLD, the frequency of *C9orf72* expansions in a Western European population was 9.98% in overall FTLD, with 18.52% in familial and 6.26% in sporadic FTLD patients [44]. Though the low frequency of *C9orf72* repeat expansions in the FTD Bulgarian cohort studied here, we could not compare it with other prevalence studies due to the small sample size.

Consistent with previous reports in which up to 15% of FTD patients develop signs of MND [46], we found that 8.5% (7/82) of our FTD patients had FTD-ALS. Furthermore, Bulgarian patients with FTD-ALS were more likely to carry a *C9orf72* repeat expansion (1/7, 14%), similar to the increased risk (33%-86%) of harbouring a *C9orf72* repeat expansion in patients with concurrent ALS and FTD or with a family history of dementia or ALS reported in the literature [14].

Screening for *C9orf72* expansion in the AD cohort (n = 37) resulted in the identification of one AD patient (2.7%) who carried a pathological expansion. This rate is greater than the previously reported mutation rates in AD cohorts (0.9–1%) [47]. This could be explained by the small size of the AD cohort and the high proportion of EOAD cases (32/37) as well as by the presence of familial/dominant cases in our AD cohort. Wallon et al. (2012) [48] showed that 2.6% of sporadic and familial cases of EOAD had *C9orf72* expansions. *C9orf72* expansions are not a common cause of clinical AD, but such expansions could nevertheless underlie a neurodegenerative process that presents with a clinical phenotype compatible with AD [49]. On one hand, the hippocampal involvement in patients with *C9orf72* expansions may mimic the clinical aspects of AD through an impact on memory dysfunction, resulting in misdiagnosed FTD [50]. On the other hand, there are several reports of the identification of a pathogenic repeat expansion in patients with cerebrospinal fluid (CSF) biomarker profiles typical of AD [48] and in autopsy-confirmed AD cases [51,52]. However, in these cases, concomitant AD that is causally unrelated to *C9orf72* expansions cannot be completely ruled out. In 2 series of 568 and 424 AD patients, no pathogenic repeat expansions were detected [53,54].

The onset of disease in our AD patient and in his family members occurred between 61 and 71 years of age. The age at onset is similar to that reported in another study of AD patients, in which identified expansion carriers experienced disease onset between 61 and 71 years of age. This suggests that disease onset within this this age range may be a characteristic feature of *C9orf72* repeat expansion carriers with a predominant amnestic syndrome [49,55].

We present four cases with *C9orf72* repeat expansions for whom the neuroimaging and neuropsychological data support the presence of various degrees of hippocampal atrophy and memory impairment of the predominantly hippocampal type (in three cases) early in the disease course. The degree of memory dysfunction in these patients varied from mild to marked; when memory dysfunction was present, it was often evident early in the disease course. It has been suggested that c9FTD cases may present with memory impairment. As discussed above, this feature was sufficiently prominent to lead to a clinical diagnosis of AD in some cases and has led to the suggestion that c9FTD cases can present with a distinct ‘amnestic profile’, a problematic differential diagnostic feature [47,50,55].

Parkinsonism appeared in some degree as an early or late clinical feature in all four *C9orf72* cases. Previous neuropathological studies have shown that nigrostriatal involvement is common in cases with *C9orf72* repeat expansion and that such involvement can be clearly distinguished from Parkinson disease–related mechanisms by the presence of p62-positive inclusions and the absence of α-synuclein–positive Lewy bodies or Lewy neurites [56]. The simultaneous presence of MND and extrapyramidal features in the same individual, although reported in the literature, is rare [57]. The recent observations broaden the spectrum of clinical phenotypes associated with *C9orf72* and suggest the existence of a novel *C9orf72*-positive ALS-parkinsonism (case 2) nosological entity [58] that may be driven by an increased lesion load in extramotor areas, including the nigrostriatal system [59]. In Case 2 with FTD-ALS, the parkinsonian signs were present at the onset of the disease, and the ALS signs emerged within the first two years of the disease, consistent with previous reports [13,60]. Early parkinsonian signs were not observed in the other 6 non-*C9orf72* cases.

We also report a case of PSP with behavioural features consistent with bvFTD. Few studies have reported *C9orf72* expansions in patients with PSP. To the best of our knowledge, only 2 studies have identified PSP cases with *C9orf72* expansions. Lesage et al. (2013) [9] found one *C9orf72* expansion in 123 clinically diagnosed PSP patients, and Origione et al. (2013) [61] reported one *C9orf72* repeat expansion among 12 clinically diagnosed PSP patients. The clinical phenotype associated with *C9orf72* repeat expansions may be broader than originally thought and may possibly involve extramotor neuronal structures such as the basal ganglia, the cerebellum, and/or the brainstem nuclei [15].

Case 3 with PSP was scored as a sporadic case. The parents of this patient died at 58 years of age. The negative family history could be explained by early death of family members carrying the expansion, non-paternity or a lack of medical information in previous generations. Another possible explanation of the occurrence of the repeat expansion in apparently sporadic cases is reduced penetrance of the repeat expansion in *C9orf72* or de novo expansions [1,2]. Interestingly, Case 4 with AD had a cousin with a diagnosis of multiple sclerosis (MS); no DNA from the cousin was available for genotyping of a possible *C9orf72* expansion. Although *C9orf72* expansions do not appear to play a major role in MS pathogenesis, Lorefice et al. (2015) [62] found *C9orf72* pathogenic repeat expansions in 6/1014 MS patients (0.6%). It is also evident that the MS-ALS cases with a *C9orf72* repeat expansion described by Ismail et al. (2013) [63] are characterized by more rapid progression of the disease than occurs in patients with pure *C9orf72*-ALS, raising the hypothesis that penetrance and progression of the *C9orf72* expansion may be affected by MS-associated neurodegeneration or neuroinflammation.

Case 2, who had a diagnosis of FTD-ALS, had family members with pure ALS. The phenotypic heterogeneity in this family might be associated with germline or somatic variations in the repeat sizes of the mutated and/or wild-type *C9orf72* alleles; additional unidentified genetic modifiers might also be involved [10,64].

At a clinical level, frontal atrophy was detected in three cases, two of which had subtle atrophy. Studies have shown that the degree of atrophy in C9FTD patients is occasionally much more subtle than expected based on the clinical phenotype. Very mild asymmetric frontal/frontotemporal atrophy was seen in the bvFTD and FTD-ALS cases. *C9orf72* expansion has been associated primarily with relatively symmetrical (bilateral) atrophy that is most prominent in the frontal and temporal lobes and in the insula. The MRI of at least two patients (Cases 3 and 4) did not show the frontotemporal atrophy pattern that is typical of FTD. In the early stages of the disease, MTLA was pronounced in 2 of the 4 cases; posterior atrophy was detected in the AD index. Some studies have shown that diffuse cortical atrophy that includes anterior as well as posterior structures and subcortical involvement may a represent unique feature associated with *C9orf72* repeat expansions. Another study broadened the *C9orf72* phenotype and placed hippocampal sclerosis dementia with amnesic phenotype and focal hippocampal atrophy in the FTD spectrum [47, 65, 66].

None of our PPA subtype cases (N = 17) were found to have a *C9orf72* repeat expansion, similar to the findings reported in several previous studies [13,54] and in contrast to the results of studies by other groups who reported cases presenting with either progressive non-fluent aphasia or semantic dementia [12,17,18].

Interestingly, we found early-stage writing errors without aphasia in two cases with *C9orf72* expansion. To the best of our knowledge, our study is the first study in which early-stage writing errors without aphasia have been found in FTD patients with *C9orf72* expansion. Previously, little attention was paid to writing impairment. One explanation for this could be the lack of sophisticated written language analysis that has been conducted in most cases with identified *C9orf72* expansions. Furthermore, detailed handwriting language assessment can only be performed in patients without severe motor weakness and dysfunction. Agraphia is classified into several types–pure agraphia, aphasic agraphia, agraphia with alexia, apraxic agraphia, and spatial agraphia [67]. Two of the patients with *C9orf72* expansion (cases 1 and 2) in this study were classified as having pure agraphia. Both patients had normal language development with no history of problems in reading or writing. At the time of examination, the severity of cognitive impairments among the patients with *C9orf72* expansion ranged from mild cognitive impairment (cases 2 and 3) to mild dementia (cases 1 and 4). The errors were neither apraxic nor spatial. Writing errors were characterized by omissions, transpositions, insertions, substitutions, repetition, and jargonagraphia. We found agraphia on the basis of dictation and written confrontational naming, whereas oral confrontation naming was relatively preserved. The patients showed greater disability in written confrontation naming than in dictation.

**Fig 4:**
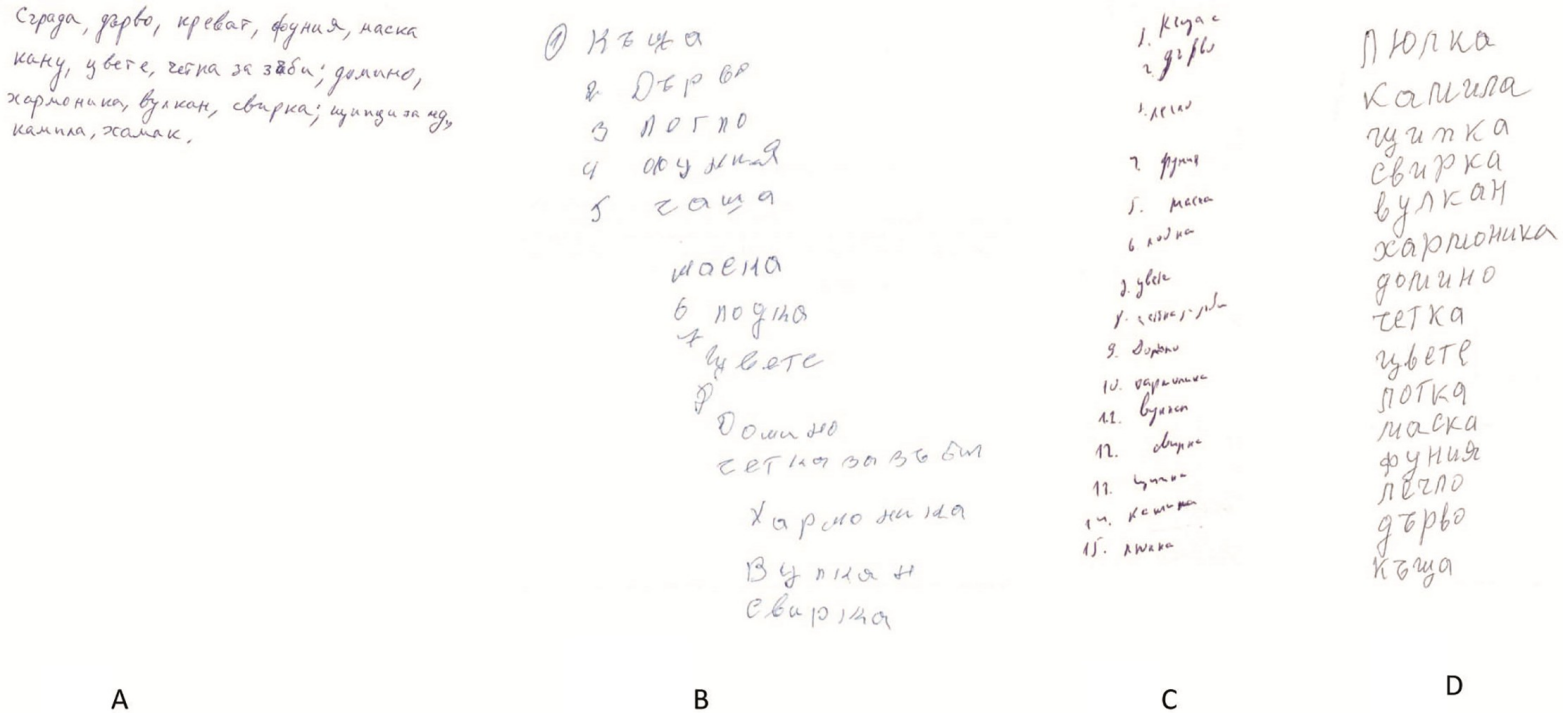
Characteristics of written confrontation naming in various patients in the Bulgarian dementia cohort. A. Correct writing naming in a patient with nf-PPA (MMSE = 22). B. Correct written naming with apraxic handwriting in a patient with CBS (MMSE = 25). C. Micrographia in a patient with FTD-PSP and *C9orf72* expansion (MMSE = 25). D. Correct writing naming in a patient with bvFTD without *C9orf72* expansion (MMSE = 20).

Analysis of writing dysfunction in the context of neurodegenerative disorders indicates that it is often more closely related to general cognitive or executive dysfunction than to language dysfunction, as shown by the results of a recent study of FTD patients with MAPT mutations [68]. Agraphia in FTD was previously described in terms of aphasic agraphia in PPA [69], surface dysgraphia in semantic dementia [70], jargonagraphia in non-fluent PPA [71], and allographic agraphia [72]. Fig 4 shows the characteristics of written confrontation naming in different neurodegenerative disorders found in the Bulgarian dementia cohort–a nf-PPA case (correct writing), a patient with CBS (correct written naming with apraxic handwriting), a bvFTD case without C9orf72 expansion (correct writing), and an FTD-PSP case with *C9orf72* expansion (correct writing with micrographia).

Detailed language studies of the group of patients with bv-FTD and bv-FTD+ALS did not reveal agraphia in the early stage of the disease. Furthermore, patients with PPA, particularly non-fluent PPA, in which early agraphia is a characteristic feature, usually present with agrammatic oral and written spontaneous speech. Acquired agraphia is usually evident in spontaneous narrative writing (omission of verbs and other functional words, morpheme omissions and substitutions, and phoneme and verbal paragraphias). We present an example of a patient with nf-PPA (MMSE = 22) with impairment in spontaneous narrative writing but correct written confrontation naming (Fig 4).

It is worth noting that acquired writing disorders in Bulgarian patients are relatively rare as an isolated symptom and are mainly observed in the context of aphasia. The written picture-naming task is suggested to be a relatively easy written task for Bulgarian patients. This could be explained by the specificity of the Bulgarian transparent language system (direct grapheme-phoneme correspondence) [73].

In Western countries, writing errors in ALS were first documented by Ferguson and Boller in 1977 [74]. However, subsequent descriptions of writing errors after the late 1990s in ALS patients focused on progressive aphasia combined with MND/ALS [75]. Language-dominant FTD(PPA)-MND is associated with bulbar onset ALS [76], suggesting that a common cortical degenerative process causes the language abnormalities in PPA and tongue and bulbar muscle weakness in ALS [18].

Furthermore, some Japanese investigators reported that ALS patients frequently had agraphia without aphasia regardless of whether or not they had dementia [75,77,78,79]. An autopsied case with progressive agraphia and ALS with dementia showed marked degeneration of the left middle frontal gyrus, including Exner's area (graphemic/motor frontal area) [80]. A recent clinico-anatomical study of writing deficits in PPA patients demonstrated two distinct patterns of spelling errors with different neural substrates: a phoneme-to-grapheme route and a whole-word route. The first pattern is seen primarily in patients with nf-PPA; the associated areas of cortical atrophy are the inferior frontal gyrus and the supramarginal gyrus of the inferior parietal lobule. The second pattern is observed predominantly in semantic PPA; the associated areas of atrophy are the fusiform gyrus and the temporal pole (ventral pathway) [81]. Taken together, these findings suggest that writing errors are closely associated with the language-related frontotemporal lobe [76, 82].

Our results indicate that writing errors may occur in FTD patients with *C9orf72* expansions and that these errors are not merely the consequences of dementia/aphasia. The finding suggests that the writing errors observed in this context may occur due to selective involvement of extra-motor regions rather than due to diffuse brain dysfunction [75]. It remains unclear whether writing errors are an early sign of the development of dementia in FTD and ALS patients with *C9orf72* expansions. The presence of writing errors is a common finding in patients with bulbar onset ALS in the Japanese population; however, the frequency of *C9orf72* expansions in the Japanese population is low [80,83]. It is possible that this association may be mediated by an underlying pathology rather than a mutation effect. *C9orf72*-related neurodegeneration is a clinically and pathologically heterogeneous syndrome that is characterized by a combination of TDP-43 proteinopathy and superimposed extramotor p62-positive, TDP-43-negative pathology. The distribution and severity of the latter pathology is likely to govern the presence of various specific cognitive and motor impairments. We suggest that a specific spread of the neuropathological process through the language-related zones and not language variants of FTD might be the basis of early writing errors in FTD/ALS [56].

In one patient (Case 1) with a clinical diagnosis of bvFTD, subtle behavioural changes were observed several years before dementia was diagnosed; this is consistent with the results of previous studies and suggests that early subtle behavioural changes are a distinguishing feature of disease associated with *C9orf72* expansions [84]. The same patient had early psychosis and hallucinations, both of which are reported to be prominent features of pathologies associated with this mutation [11]. There are behavioural signs that, taken together, might predict the presence of *C9orf72* repeat expansions; these include psychotic symptoms, complex repetitive behaviours linked to a mono-delusion or with an obsessive-compulsive quality, and absence of sweet food preference [11]. All the patients except the clinical AD case had early prominent behavioural changes based on FBI (Table 3).

The small number of cases and the use of a cohort selected based on the availability of DNA samples are the main limitations of this study. Despite detailed neuropsychological examination of the patients in this cohort, including the *C9orf72* repeat expansion carriers, we did not fully clarify the mechanisms underlying writing errors. Furthermore, the small number of cases prevents us from drawing strong conclusions regarding the importance of agraphia in the clinical and neuropsychological features of *C9orf72* repeat expansions.

In conclusion, this study represents the first genetic screening of *C9orf72* repeat expansions in a Bulgarian dementia cohort. The *C9orf72* repeat expansion does not appear to be a common cause of FTD or related disorders. This report confirms the notion that pathogenic *C9orf72* expansions underlie a broad spectrum of neurodegenerative phenotypes. Some specific clinical, neuropsychological, neuropsychiatric and neuroimaging features of pathogenic *C9orf72* expansions were identified. This has important implications for clinicians, who should consider genetic testing of patients with neurodegenerative disorders. The presence of relatively isolated agraphia in two cases with *C9orf72* expansions is a strong motivation to provide detailed and sophisticated oral and written language assessments that can be used to more precisely characterize the early cognitive deficits associated with these neuropathologically heterogeneous conditions.

## Abbreviations

AD: Alzheimer’s disease
ALS: Amyotrophic lateral sclerosis
BDAE: Boston Diagnostic Aphasia Examination
bvFTD: behavioral variant FTD
CBD: corticobasal degeneration
CERAD: Consortium to Establish a Registry for Alzheimer's Disease
EOAD: Eearly onset Alzheimer’s disease
FCSRT: Free and Cued Selective Reminding Test
FTD: Frontotemporal dementia
IST: Isaacs Set Test
LOAD: Late onset Alzheimer’s disease
MND: motor neuron disease
nf-PPA: non-fluent primary progressive aphasia
PSP: progressive supranuclear paralysis
ROCFT: Rey-Osterrieth Complex Figure Test
TMT: Trial Making Test
WMH: White matter hyperintensities
